# Carbamazepine promotes surface expression of mutant Kir6.2-A28V ATP-sensitive potassium channels by modulating Golgi retention and autophagy

**DOI:** 10.1016/j.jbc.2022.101904

**Published:** 2022-04-06

**Authors:** Ching-Han Lin, Yu-Chi Lin, Shi-Bing Yang, Pei-Chun Chen

**Affiliations:** 1 Division of Endocrinology and Metabolism, Department of Internal Medicine, National Cheng Kung University Hospital, Tainan, Taiwan; 2 Department of Physiology, College of Medicine, National Cheng Kung University, Tainan, Taiwan; 3 Institute of Biomedical Sciences, Academia Sinica, Taipei, Taiwan; 4 Graduate Institute of Basic Medicine, College of Medicine, National Cheng Kung University, Tainan, Taiwan

**Keywords:** ATP-sensitive potassium channel, inwardly rectifying potassium channel 6.2, persistent hyperinsulinemic hypoglycemia of infancy, sulfonylurea receptor, AMPK, AMP-activated protein kinase, BSA, bovine serum albumin, Carb, carbamazepine, cDNA, complementary DNA, co-IP, coimmunoprecipitation, CQ, chloroquine, dPBS, Dulbecco's PBS, ER, endoplasmic reticulum, ERAD, ER-associated protein degradation, HA, hemagglutinin, HEK293, human embryonic kidney 293 cell line, hKir6.2, human Kir6.2, hSUR1, human SUR1, IgG, immunoglobulin G, K_ATP_, ATP-sensitive potassium channel, mTOR, mammalian target of rapamycin, PHHI, persistent hyperinsulinemic hypoglycemia of infancy, PLA, proximity ligation assay, RKR, arginine–lysine–arginine, RRID, Research Resource Identifier, SUR1, sulfonylurea receptor 1, UPR, unfolded protein response

## Abstract

Pancreatic β-cells express ATP-sensitive potassium (K_ATP_) channels, consisting of octamer complexes containing four sulfonylurea receptor 1 (SUR1) and four Kir6.2 subunits. Loss of K_ATP_ channel function causes persistent hyperinsulinemic hypoglycemia of infancy (PHHI), a rare but debilitating condition if not treated. We previously showed that the sodium-channel blocker carbamazepine (Carb) corrects K_ATP_ channel surface expression defects induced by PHHI-causing mutations in SUR1. In this study, we show that Carb treatment can also ameliorate the trafficking deficits associated with a recently discovered PHHI-causing mutation in Kir6.2 (Kir6.2-A28V). In human embryonic kidney 293 or INS-1 cells expressing this mutant K_ATP_ channel (SUR1 and Kir6.2-A28V), biotinylation and immunostaining assays revealed that Carb can increase surface expression of the mutant K_ATP_ channels. We further examined the subcellular distributions of mutant K_ATP_ channels before and after Carb treatment; without Carb treatment, we found that mutant K_ATP_ channels were aberrantly accumulated in the Golgi apparatus. However, after Carb treatment, coimmunoprecipitation of mutant K_ATP_ channels and Golgi marker GM130 was diminished, and K_ATP_ staining was also reduced in lysosomes. Intriguingly, Carb treatment also simultaneously increased autophagic flux and p62 accumulation, suggesting that autophagy-dependent degradation of the mutant channel was not only stimulated but also interrupted. In summary, our data suggest that surface expression of Kir6.2-A28V K_ATP_ channels is rescued by Carb treatment *via* promotion of mutant K_ATP_ channel exit from the Golgi apparatus and reduction of autophagy-mediated protein degradation.

Pancreatic ATP-sensitive potassium (K_ATP_) channels are composed of four Kir6.2 subunits and four regulatory sulfonylurea receptor 1 (SUR1) subunits. The primary function of these channels is to sense the cytosolic ATP and ADP concentrations in pancreatic β-cells and respond by regulating cellular electrical activities. As such, K_ATP_ channels link metabolism status to membrane potential and play an essential role in regulating insulin secretion (1). Persistent hyperinsulinemic hypoglycemia of infancy (PHHI) is a rare genetic disease caused by mutations in the K_ATP_ channel subunits that has an incidence rate of about 1 in 50,000. Despite this low incidence, PHHI is one of the most important and frequent causes of severe neonatal hypoglycemia (2). The first-line treatment for PHHI is diazoxide, a K_ATP_ channel opener that can suppress insulin release. Unfortunately, however, 90% of all neonatal forms of PHHI are resistant to diazoxide, and patients with these forms of disease must undergo a near-total pancreatectomy. Recently, we have identified a *KCNJ11* mutation that causes late-onset PHHI. This nonsynonymous C83T point mutation encodes Kir6.2-A28V, and carriers of this allele exhibit diazoxide-resistant PHHI. Electrophysiological recordings revealed that K_ATP_ channels with the Kir6.2-A28V mutant have almost no current, as the mutant K_ATP_ channels fail to reach the cell surface in Kir6.2-A28V-transfected cells (3). Based on *de novo* protein structure prediction, a plausible mechanism leading to the trafficking deficit is the exposure of the RKR (arginine–lysine–arginine) motif in Kir6.2-A28V during protein folding. If the RKR motif is not properly concealed, the protein can be aberrantly retained in the endoplasmic reticulum (ER) (4).

Folding of proteins requires the assistance of molecular chaperones, often of the heat shock protein family. There are several chaperone functions of heat shock proteins that ensure the quality of mature proteins. Molecular chaperones may assist in the folding of nascent proteins, play a protective role during assembly of protein complexes, prevent misfolded proteins from aggregation, or mediate degradation of certain targets. In conditions of stress, the expression of chaperones is often increased by mechanisms that serve to maintain the cellular environment (5). When new proteins are synthesized during stressful or normal conditions, chaperones can ensure protein folding efficiency and stable conformations. Chaperones can also prevent proteins from forming abnormal aggregates. Several well-known chaperones can bind and hydrolyze ATP during their interactions with misfolded proteins; this activity improves folding efficiency and ensures quality. Some ATP-dependent chaperones can function as protein remodeling factors that target misfolded or aggregated proteins for degradation or reverse the formation of dysfunctional aggregated conformations (6). Many mutations in *ABCC8* or *KCNJ11* lead to protein misfolding that causes the loss-of-channel function and PHHI. In fact, many disease-causing K_ATP_ channel mutants have trafficking deficits and cannot reach the cell surface, probably because of actions of the cellular protein quality control system. Notably, some of these trafficking defects can be corrected by sulfonylureas, which are K_ATP_ channel blockers and a second-line treatment for type II diabetes (7).

In addition to sulfonylureas, carbamazepine (Carb; an anticonvulsant thought to target voltage-gated sodium channels) was also shown to correct K_ATP_ channel trafficking defects in PHHI (8). In this study, we investigated if Carb can rescue the trafficking deficit associated with the Kir6.2-A28V mutant. Carb treatment increased the total and surface expression of SUR1 in SUR1/Kir6.2-A28V transfected cells, according to a surface biotinylation assay. The rescue effects of Carb on the trafficking defects did involve the unfolded protein response (UPR). Simultaneously, we also found that Carb treatment impaired the autophagy-dependent protein degradation pathway, allowing the mutant K_ATP_ channels to travel through the ER to the Golgi apparatus, where mutant K_ATP_ channels interact with GM130, a Golgi matrix protein. Surprisingly, Carb treatment decreased this interaction; instead, it allowed mutant K_ATP_ channels to exit from the Golgi apparatus and traveled to the cell surface. Collectively, our results reveal novel mechanisms by which Carb rescues the trafficking defects of a mutant K_ATP_ channel.

## Results

### Carb promotes surface expression of the trafficking-defected K_ATP_ channel mutant (Kir6.2 A28V/SUR1)

**Figure 1:**
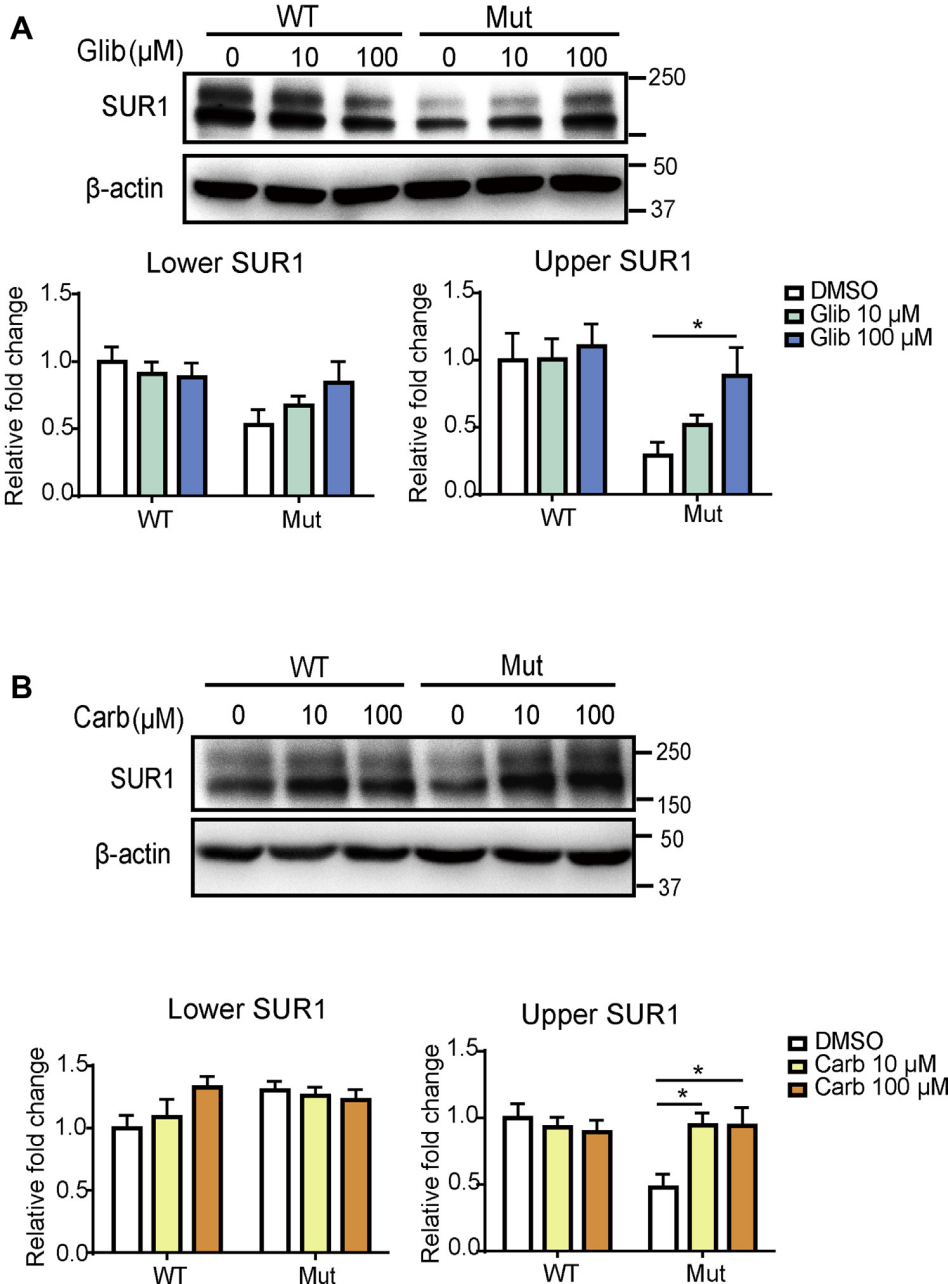
**Glibenclamide and carbamazepine (Carb) increase mature SUR1 in cells expressing mutant K**_**ATP**_**channels.***A*, *top*, representative images show the effects of Glib on SUR1 in HEK293 cells expressing WT (SUR1/Kir6.2) or mutant (SUR1/Kir6.2-A28V) K_ATP_ channels. *Bottom*, quantification of the upper (mature) and lower bands of SUR1 is shown in the *left* and *right* plots, respectively. *B*, *top*, representative images show the effects of Carb on SUR1 in HEK293 cells expressing WT or mutant K_ATP_ channels. *Bottom*, quantification of the upper (mature) and lower bands of SUR1 is shown in the *left* and *right* plots, respectively. Data are presented as the mean ± SEM; n = 8 per group; ∗*p* < 0.05, compared with DMSO-treated cells in each corresponding group; differences were evaluated using two-way ANOVA and Dunnett's post hoc test. DMSO, dimethyl sulfoxide; HEK293, human embryonic kidney 293 cell line; K_ATP,_ ATP-sensitive potassium channel; SUR1, sulfonylurea receptor 1.

We previously demonstrated that the pharmacochaperones, Carb and Glib, could rescue the trafficking defects of K_ATP_ channels containing several PHHI-causing SUR1 mutants (7, 8, 9, 10, 11). Thus, we wanted to test whether Carb and Glib could also rescue the trafficking deficits of mutant K_ATP_ channels containing Kir6.2-A28V. We first asked whether Carb and Glib could rescue the maturation and surface expression of the mutant K_ATP_ channels. WT and mutant K_ATP_ channels were expressed in human embryonic kidney 293 (HEK293) cells, and then the cells were treated with vehicle (0.1% dimethyl sulfoxide), 10 μM Glib or Carb, and 100 μM Glib or Carb. The cells were then subjected to Western blot analysis after 16 h of treatment. Both Glib and Carb had negligible effects on the WT K_ATP_ channels but dose-dependently increased the upper (mature) SUR1 band in the mutant K_ATP_ channel–expressing cells (Fig. 1*A*, *top*). We have previously shown that the upper band is the complex-glycosylated SUR1, according to its sensitivity to the peptide-N-glycosidase F (data not shown). Quantification of the upper SUR1 band showed that in 100 μM Glib-treated cells, mature SUR1 protein was significantly increased as compared with that in the vehicle-treated group (Fig. 1*A*, *bottom*). Similarly, Carb dose-dependently rescued mutant K_ATP_ channels, as the upper SUR1 band was significantly increased after 10 μM or 100 μM Carb treatment, in comparison with vehicle-treated cells (Fig. 1*B*). Although both Carb and Glib could rescue the impaired maturation of SUR1 caused by the Kir6.2-A28V mutant, Glib irreversibly inhibits K_ATP_ channels formed by Kir6.2 and SUR1 (12). Thus, we focused on Carb for further mechanistic studies in INS-1 cells.

**Figure 2:**
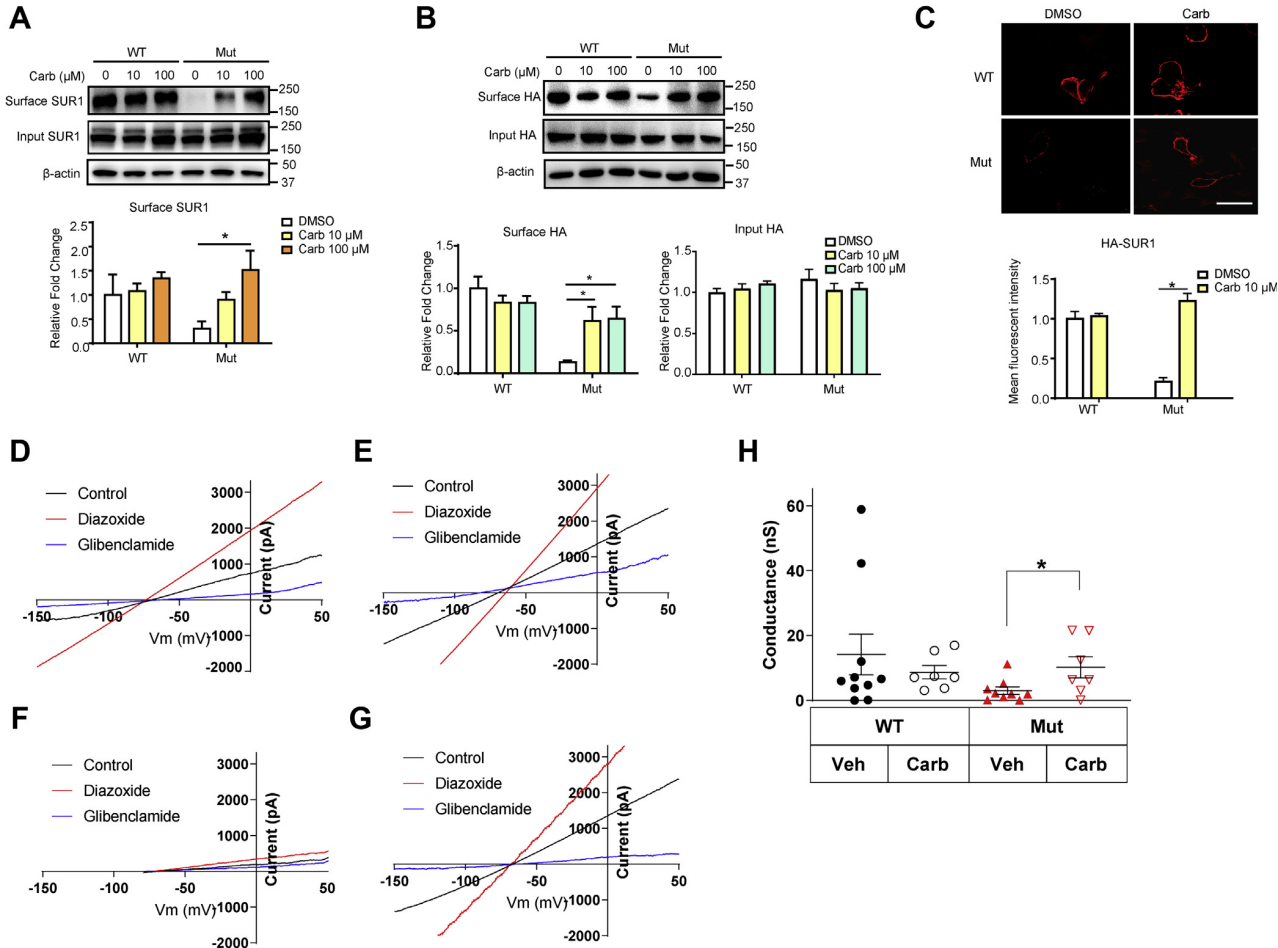
**Carbamazepine (Carb) increases surface SUR1 expression in INS-1 cells expressing mutant K**_**ATP**_**channels.***A*, *top*, representative blotting images show the surface biotinylation of SUR1 in INS-1 cells expressing WT (HA-SUR1/Kir6.2) or mutant (HA-SUR1/Kir6.2-A28V) K_ATP_ channels, after treatment with 10 μM or 100 μM Carb for 16 h. *Bottom*, quantification of surface SUR1 is shown in the plot. *B*, *top*, representative blotting images show the surface biotinylation of HA epitope in INS-1 cells expressing WT or mutant K_ATP_ channels after treatment with 10 μM or 100 μM Carb for 16 h. *Bottom*, quantification of surface and input HA epitope were shown in the plots separately. *C*, *top*, representative immunostaining images showed surface staining of HA-tagged WT and mutant K_ATP_ channels in INS-1 cells. *Bottom*, the bar graph shows the fluorescence intensity of the HA signal. Data are presented as the mean ± SEM; n = 8 per group; ∗*p* < 0.05, compared with the DMSO-treated cells in each corresponding group; differences were evaluated using two-way ANOVA and Dunnett's post hoc test. The scale bar represents 10 μm. *D*, representative WT K_ATP_ currents (*black*) elicited by a voltage ramp (0.5 V/s). The K_ATP_ currents were activated by 300 μM K_ATP_ channel opener diazoxide (*red*) and inhibited by 10 μM K_ATP_ channel blocker glibenclamide (*blue*). *E*, representative WT K_ATP_ currents from a HEK293 cell treated with 10 μM Carb overnight. *F*, representative Kir6.2 A28V K_ATP_ currents from a HEK293 cell treated with vehicle control. *G*, representative Kir6.2 A28V K_ATP_ currents from a HEK293 cell treated with 10 μM Carb overnight. *H*, summary of WT and Kir6.2 A28V K_ATP_ currents in HEK cells treated with either vehicle control or 10 μM Carb overnight. This overnight Carb treatment significantly augmented the whole-cell Kir6.2 A28V K_ATP_ currents (*t* test, ∗*p* < 0.05). DMSO, dimethyl sulfoxide; HA, hemagglutinin; HEK293, human embryonic kidney 293 cell line; K_ATP_, ATP-sensitive potassium channel; SUR1, sulfonylurea receptor 1.

To confirm that Carb treatment actually causes the mature mutant K_ATP_ channels to reach the cell surface, we performed surface biotinylation and subsequent immunoprecipitation to determine the levels of surface protein expression. We cotransfected Kir6.2-A28V with hemagglutinin (HA)-tagged SUR1 to express mutant K_ATP_ channels in rat insulinoma INS-1 cells, a rat insulinoma cell line that still maintains specific beta-cell physiology and insulin secretion (13, 14). Either 10 or 100 μM Carb treatment could increase the surface expression of SUR1 in the mutant K_ATP_ channel–expressing cells, as indicated by immunoblotting for SUR1 or HA (Fig. 2, *A* and *B*). We also performed surface immunostaining to verify the effects of Carb on the surface expression of mutant K_ATP_ channels. Indeed, INS-1 cells transfected with WT but not mutant K_ATP_ channels had strong surface signals. This observation was in agreement with a previous report (3). However, mutant K_ATP_ channel–expressing cells treated with Carb had HA-SUR1 surface expression levels comparable to those observed in cells expressing WT K_ATP_ channels (Fig. 2*C*). Finally, to confirm whether the increased surface expression of the K_ATP_ channels containing the mutant Kir6.2-A28V, we performed whole-cell patch-clamp recording in HEK293 cells transfected with either SUR1/Kir6.2 (WT) or SUR1/Kir6.2-A28V. Our results (Fig. 2, *D*–*H*) showed that the Carb pretreatment could augment the mutant but not WT K_ATP_ currents, further supporting the role of Carb in promoting mutant K_ATP_ channel trafficking.

### Carb prevents Kir6.2-A28V and SUR1 from becoming trapped in the Golgi complex

**Figure 3:**
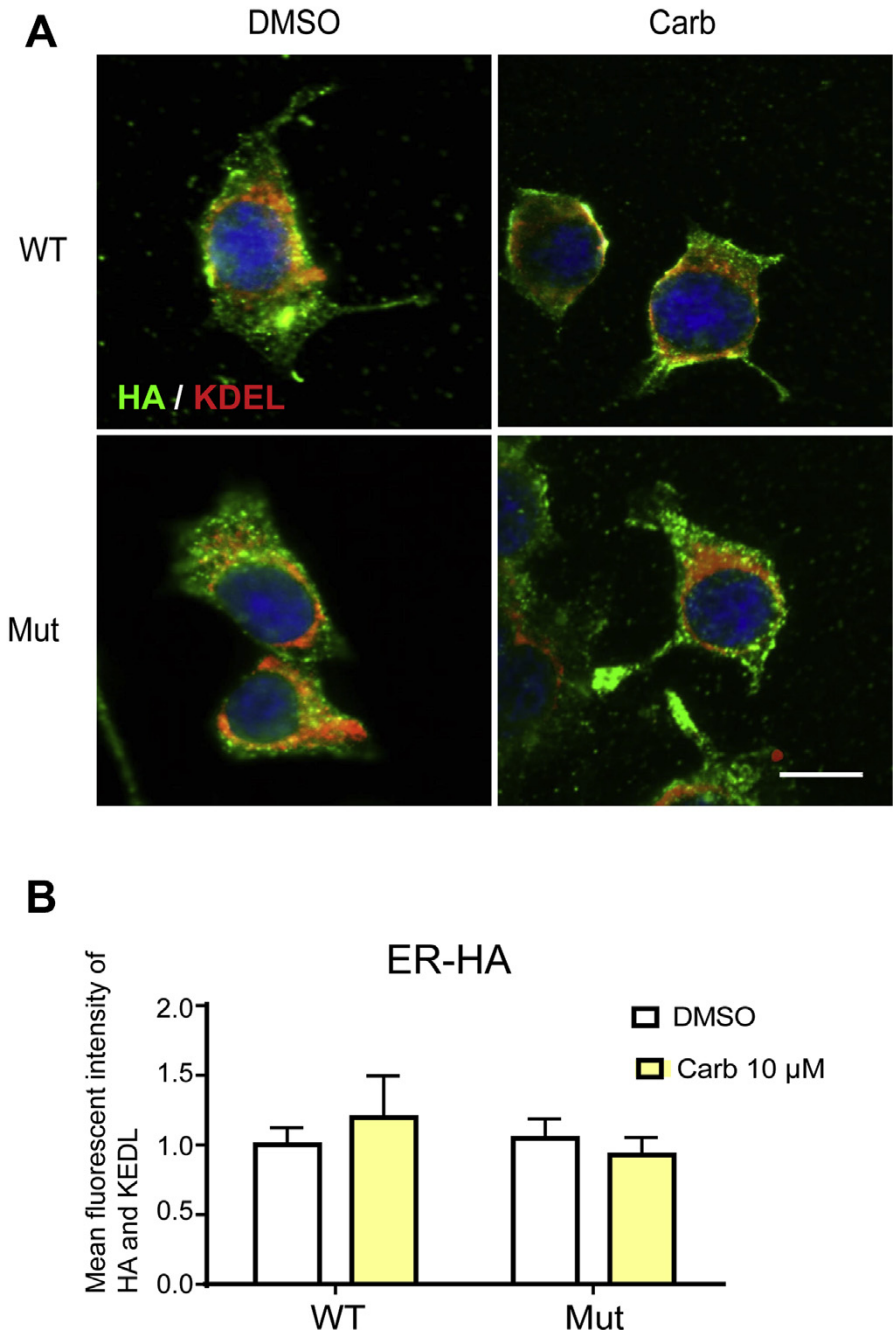
**Mutant K**_**ATP**_**channels are not trapped in the ER.***A*, *top*, representative immunostaining results show the colocalization of HA and KDEL in transfected INS-1 treated with 10 μM Carb treatment for 16 h. *B*, *bottom*, the bar graph shows the mean fluorescence intensity of colocalized HA (*green*) and KDEL (*red*) signals. Data are presented as the mean ± SEM; n = 5 per group; the scale bar represents 10 μm. Carb, carbamazepine; ER, endoplasmic reticulum; HA, hemagglutinin; K_ATP_, ATP-sensitive potassium channel.

**Figure 4:**
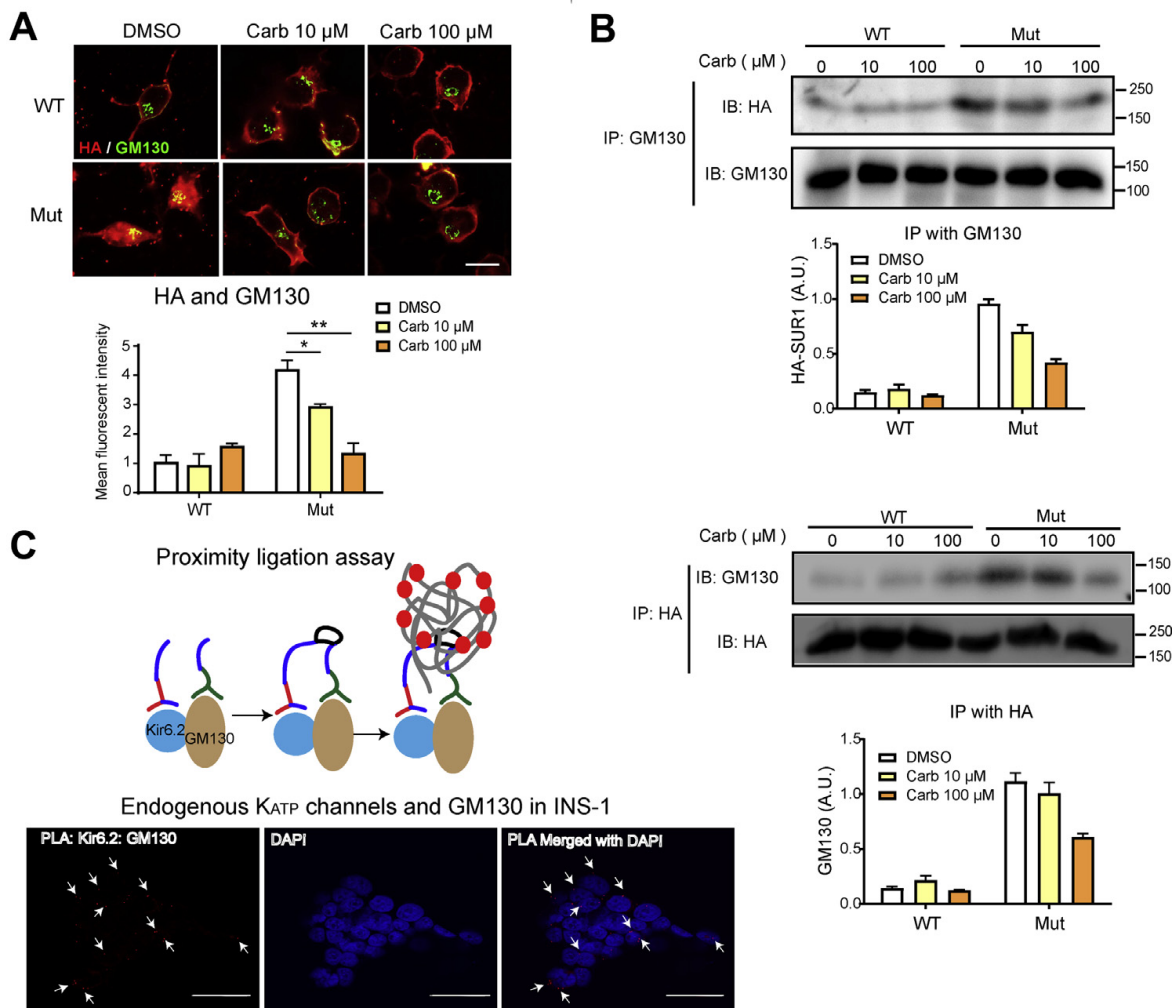
**Mutant K**_**ATP**_**channels colocalize and interact with Golgi matrix protein GM130.***A*, *top*, representative immunostaining results show colocalization of HA (*green*) and GM130 (*red*) in INS-1 cells expressing WT (HA-SUR1/Kir6.2) or mutant (HA-SUR1/Kir6.2-A28V) K_ATP_ channels, upon 10 or 100 μM Carb treatment for 16 h. *Bottom*, the bar graph shows the mean fluorescence intensity for colocalized HA and GM130. Data are presented as the mean ± SEM; n = 5 per group; ∗*p* < 0.05, ∗∗*p* < 0.01 compared with the DMSO-treated cells in each corresponding group; differences were evaluated using two-way ANOVA and Dunnett's post hoc test. The scale bar represents 10 μm. *B*, *top*, proteins were pulled down by agarose beads conjugated with GM130 antibody, followed by immunoblotting with HA and GM130 antibodies. Cells had been treated with 10 μM or 100 μM Carb treatment for 16 h. Quantification of HA-SUR1 pulled down by GM130-conjugated agarose beads is shown in the plot. *Bottom*, representative images show the interaction between GM130 and the HA epitope. *Top*, proteins were pulled down by agarose beads conjugated with HA antibody, followed by immunoblotting using HA and GM130 antibodies. Cells had been treated with 10 μM or 100 μM Carb treatment for 16 h. Representative images show the interaction between GM130 and the HA epitope. Quantification of GM130 pulled down by HA-SUR1-conjugated agarose beads is shown in the plot. Data are presented as the mean ± SEM; n = 5 per group; ∗*p* < 0.05, ∗∗*p* < 0.01 compared with the DMSO-treated cells in each corresponding group; differences were evaluated using two-way ANOVA and Dunnett's post hoc test. Data are presented as the mean ± SEM; n = 4 per group; ∗*p* < 0.05, ∗∗*p* < 0.01 compared with the DMSO-treated cells in each corresponding group; differences were evaluated using two-way ANOVA and Dunnett's post hoc test. *C*, *top*, schematic representation of proximal ligation assay (PLA). *Bottom*, fluorescence images from PLA assay using Kir6.2 and GM130 antibodies in INS-1 cells. Red fluorescence signals indicate Kir6.2–GM130 contact sites (*white arrows*) observed with confocal microscopy under 100× object and counterstained with DAPI (*blue*) for nuclei. The scale bar represents 30 μm. Carb, carbamazepine; API, 4′,6-diamidino-2-phenylindole; DMSO, dimethyl sulfoxide; HA, hemagglutinin; K_ATP_, ATP-sensitive potassium channel; SUR1, sulfonylurea receptor 1.

K_ATP_ channels containing Kir6.2-A28V fail to reach the cell surface, causing a significant reduction in whole-cell K_ATP_ currents (Fig. 2) (3). We first determined the cellular distributions of WT and mutant K_ATP_ channels in INS-1 cells transiently transfected with HA-tagged SUR1 and WT Kir6.2 or mutant Kir6.2-A28V by costaining for the protein and organelle markers. A *de novo* protein structure simulation suggested that Ala to Val mutation at position 28 would rearrange the RKR ER-retention motif (3, 4), meaning that the mutant K_ATP_ channels might remain in the ER (4). INS-1 cells expressing HA-tagged SUR1 and WT Kir6.2 or mutant Kir6.2-A28V were treated with vehicle or 10 μM Carb, followed by immunofluorescence staining with HA and KEDL (C-terminal tetrapeptide ER retention signal) antibodies. Unexpectedly, we did not observe any abnormal accumulation of mutant K_ATP_ channels in the ER (Fig. 3). It has been well established that correct trafficking and surface expression of the K_ATP_ channel complex is under the control of a tripeptide ER retention signal RKR (4), present in both SUR1 and Kir6.2. During channel assembly in the ER, SUR1 and Kir6.2 associate with one another, shielding the ER retention signal to allow the channel complex to exit the ER and traffic to the cell surface. As the channel complex travels through the medial Golgi, the glycosylation pattern is further modified to give rise to the complex glycosylated proteins. We then wondered if mutant K_ATP_ channels could exit the ER, later becoming trapped in the Golgi apparatus. Indeed, more mutant K_ATP_ channels colocalized with GM130, a *cis*-Golgi matrix protein, suggesting that the mutant K_ATP_ channels containing Kir6.2-A28V subunits accumulated in the Golgi after exiting the ER (Fig. 4*A*). Furthermore, we performed coimmunoprecipitation (co-IP) experiments using anti-HA and anti-GM130 antibodies. We found that anti-GM130 pulled down more Kir6.2-A28V mutant proteins than WT, further strengthening our conclusion that the mutant K_ATP_ channels were stuck in the Golgi complex (Fig. 4*B*). Upon Carb treatment, the fluorescence signal for mutant K_ATP_ channels was largely decreased in the Golgi and increased at the plasma membrane (Fig. 4*A*, *top*); quantification of the colocalization signal in the Golgi further supported the conclusion that Carb treatment can prevent the accumulation of mutant channels in the Golgi (Fig. 4*A*, *bottom*). Anti-GM130 also pulled down fewer mutant proteins from the Carb-treated mutant-expressing cells (Fig. 4*B*, *top*); quantification of the HA-SUR1 in the coimmunoprecipitate pulled down by GM130-cojugated agarose beads displayed Carb treatment dose-dependently reduces the interaction of mutant K_ATP_ channels in the Golgi (Fig. 4*B*, *top*). Similar results were obtained when we pulled down HA-tagged proteins and immunoblotted them with GM130 antibody (Fig. 4*B*, *bottom*); the bar graph for GM130 in the coimmunoprecipitate was dose-dependently decreased upon Carb treatment (Fig. 4*B*, *bottom*). Furthermore, we employed an immunoblotting/molecular imaging method known as the proximal ligation assay (PLS) in INS-1 cells (Fig. 4*C*, *top*). Two primary antibodies were used to target Kir6.2 and GM130, followed by secondary antibodies containing PLA probes. After amplification of DNA cycle, fluorescent-labeled oligonucleotide binds to amplified DNA, resulting the red fluorescence as bright spots, demonstrating endogenous K_ATP_ channels in GM130 (Fig. 4*C*, *bottom*).

**Figure 5:**
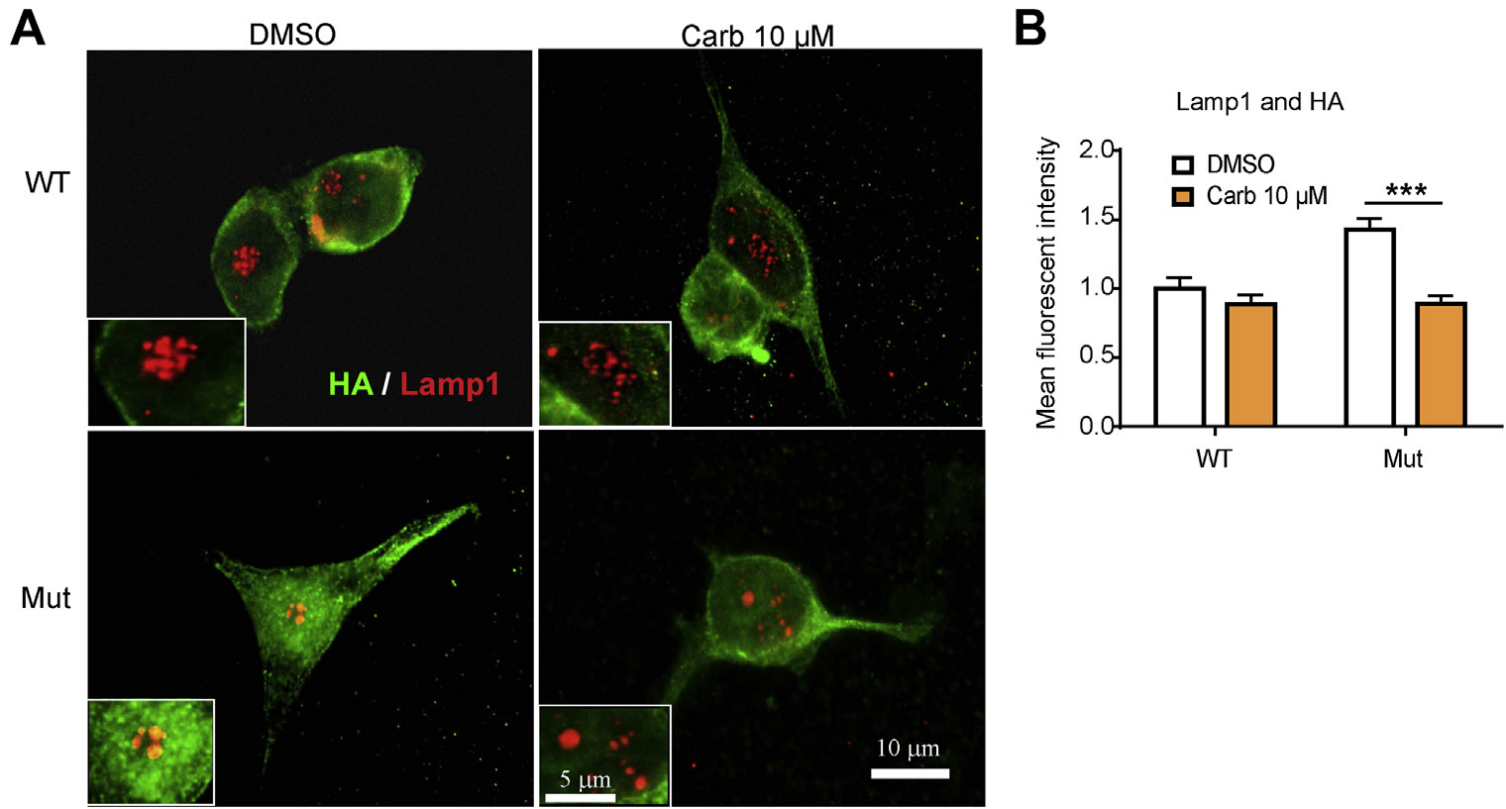
**Carbamazepine (Carb) decreases the colocalization of mutant K**_**ATP**_**channels with lysosomes.***A*, representative immunostaining images show the distribution of HA (*green*) and LAMP1 (*red*) in INS-1 cells expressing WT (HA-SUR1/Kir6.2) or mutant (HA-SUR1/Kir6.2-A28V) K_ATP_ channels, upon 10 μM Carb treatment for 16 h. *B*, the bar graph shows the mean fluorescence intensity for colocalization of HA and GM130. Data are presented as the mean ± SEM; n = 4 per group; ∗∗∗*p* < 0.005 compared with the DMSO-treated cells in each corresponding group; differences were evaluated using two-way ANOVA and Dunnett's post hoc test. The scale bar represents 10 μm. DMSO, dimethyl sulfoxide; HA, hemagglutinin; K_ATP,_ ATP-sensitive potassium channel; LAMP1, lysosomal-associated membrane protein 1; SUR1, sulfonylurea receptor 1.

Besides finding mutant K_ATP_ channels in the Golgi complex, we also observed colocalization of Kir6.2-A28V with LAMP1 (lysosomal-associated membrane protein 1), a lysosome marker, suggesting the mutant channels failed to mature in the Golgi complex and were diverted to the lysosome for degradation. Importantly, Carb treatment reduced the colocalization with lysosomes, suggesting that the degradation of mutant K_ATP_ channels had been attenuated (Fig. 5, *A* and *B*).

### Carb treatment does not induce known ER protein quality control transcriptional responses

**Figure 6:**
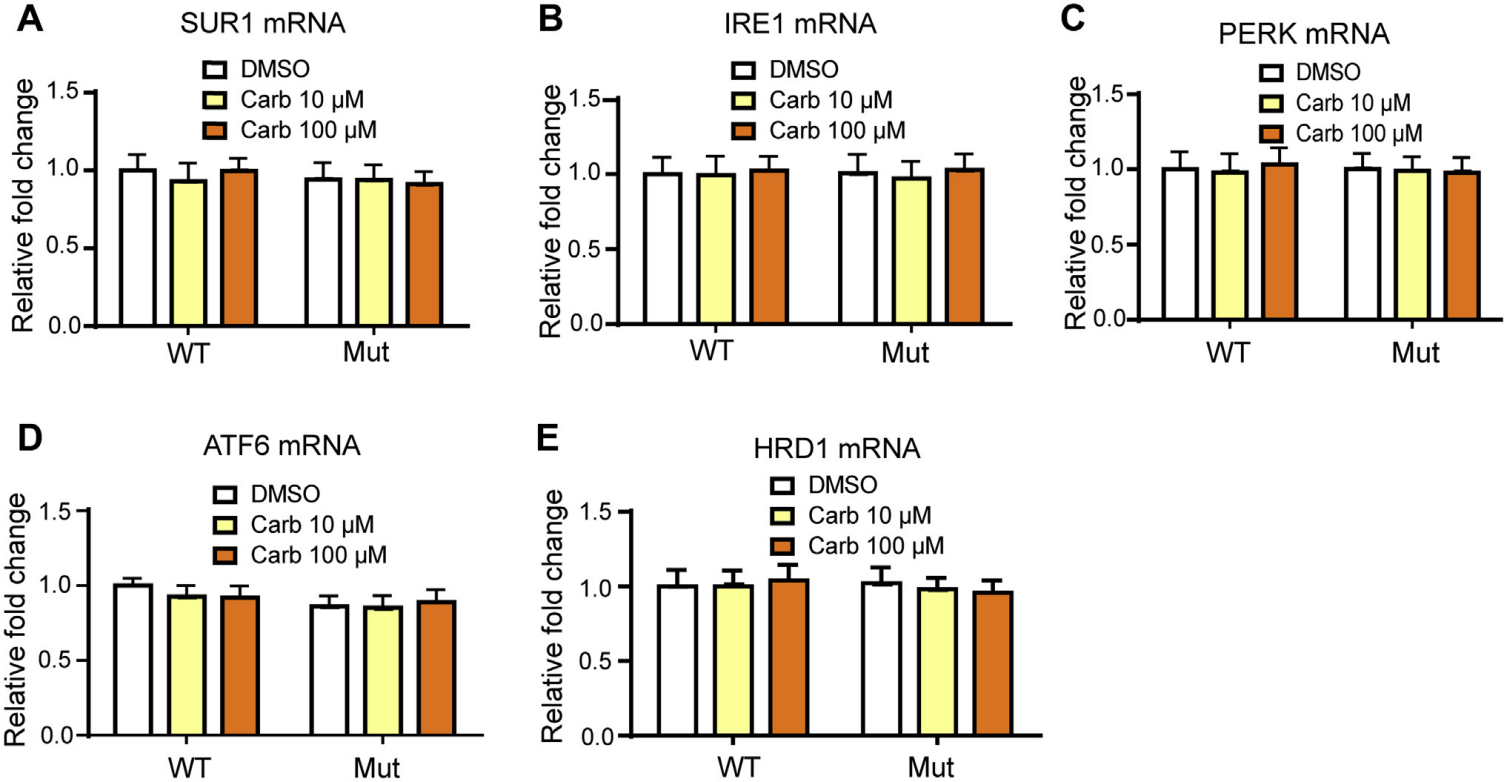
**The carbamazepine (Carb) rescue effect does not involve UPR or ERAD.***A*, relative expression levels of SUR1 mRNA in transfected HEK293 treated with 10 μM or 100 μM Carb for 16 h. The relative expression of key UPR sensors (*i.e.*, IRE1, PERK, ATF6) in INS-1 cells expressing WT (HA-SUR1/Kir6.2) or mutant (HA-SUR1/Kir6.2-A28V) K_ATP_ channels, after 10 μM Carb treatment for 16 h. Data are shown in (*B*) IRE1, (*C*) PERK, and (*D*) ATF6. *E*, the relative expression of ERAD sensor, HRD1, was also not altered. Data are presented as the mean ± SEM; n = 9 per group; no results achieved significance when compared with DMSO-treated cells in each corresponding group; differences were evaluated using two-way ANOVA and Dunnett's post hoc test. ATF6, activating transcription factor 6; DMSO, dimethyl sulfoxide; ERAD, endoplasmic reticulum–associated protein degradation; HEK293, human embryonic kidney 293 cell line; IRE1, inositol-requiring enzyme 1; K_ATP_, ATP-sensitive potassium channel; PERK, protein kinase RNA-like endoplasmic reticulum kinase; SUR1, sulfonylurea receptor 1; UPR, unfolded protein response.

To investigate if the rescue effects of Carb on mutant K_ATP_ channels act on ER-related protein quality control responses, we measured mRNA expression levels of SUR1 and key proteins in the UPR and ER-associated protein degradation (ERAD) pathways. The UPR is coordinately regulated by the three major ER stress sensors, inositol-requiring enzyme 1α (IRE1α), protein kinase RNA-like endoplasmic reticulum kinase (PERK), and activating transcription factor 6 (ATF6) (15). The activation of all three proximal sensors results in a reduction in the amount of new protein translocated into the ER lumen, increasing ERAD and promoting the transcription of endogenous chaperones (16). INS-1 cells were treated with tunicamycin at 1.25 μg/ml or dimethyl sulfoxide for 12 h, harvested RNA, and subjected quantitative PCR for UPR and ERAD genes to serve as the positive control. Tunicamycin treatment activated the UPR and ERAD pathways (Fig. S1). However, Carb did not affect SUR1 mRNA levels in WT-transfected or Kir6.2-A28V-transfected cells (Fig. 6*A*). Next, we examined the mRNA expression levels of IRE1α, PERK, and ATF6α. WT and mutant K_ATP_ channel–expressing cells with or without Carb treatments did not show any differential expression of these proteins (Fig. 6, *B*–*D*). With regard to ERAD, the mRNA expression of HRD1 was not different among cells expressing WT and mutant K_ATP_ channels, either in the presence or the absence of Carb treatment (Fig. 6*E*).

### Carb treatment attenuates autophagy-dependent protein degradation

**Figure 7:**
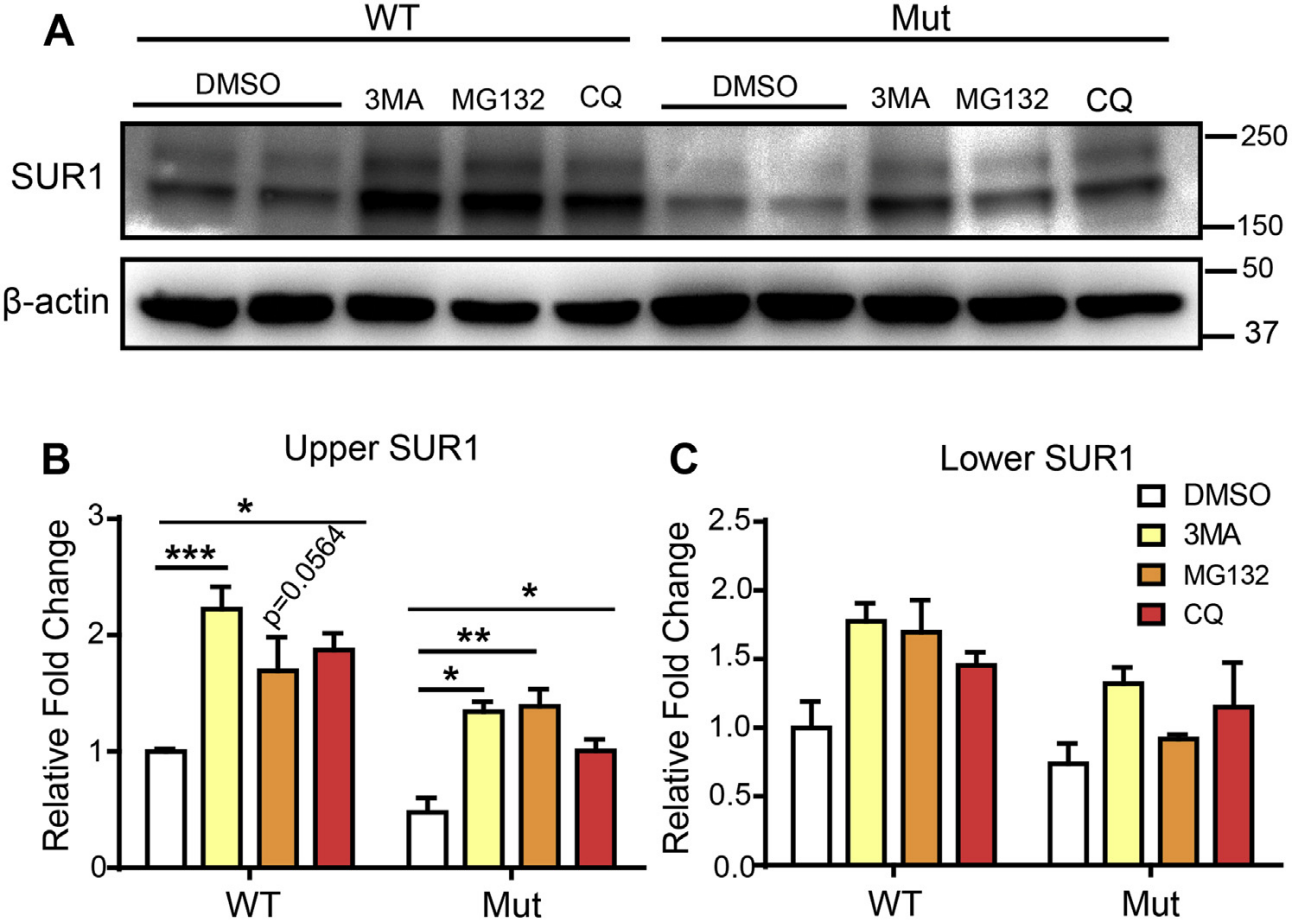
**Autophagy blocker and proteasome inhibitor increase the levels of mature SUR1 in mutant K**_**ATP**_**channels.***A*, representative immunoblotting images show the levels of SUR1 in INS-1 cells expressing WT (HA-SUR1/Kir6.2) or mutant (HA-SUR1/Kir6.2-A28V) K_ATP_ channels, after treatment with 3MA, MG132, and chloroquine (CQ). Quantification of upper (mature) SUR1 is shown in (*B*) and lower SUR1 is shown in (*C*) Data are presented as the mean ± SEM; n = 3 per group; ∗*p* < 0.05, ∗∗*p* < 0.01, ∗∗∗*p* < 0.005 compared with DMSO-treated cells in each corresponding group; differences were evaluated using two-way ANOVA and Dunnett's post hoc test. DMSO, dimethyl sulfoxide; HA, hemagglutinin; K_ATP_, ATP-sensitive potassium channel; SUR1, sulfonylurea receptor 1.

**Figure 8:**
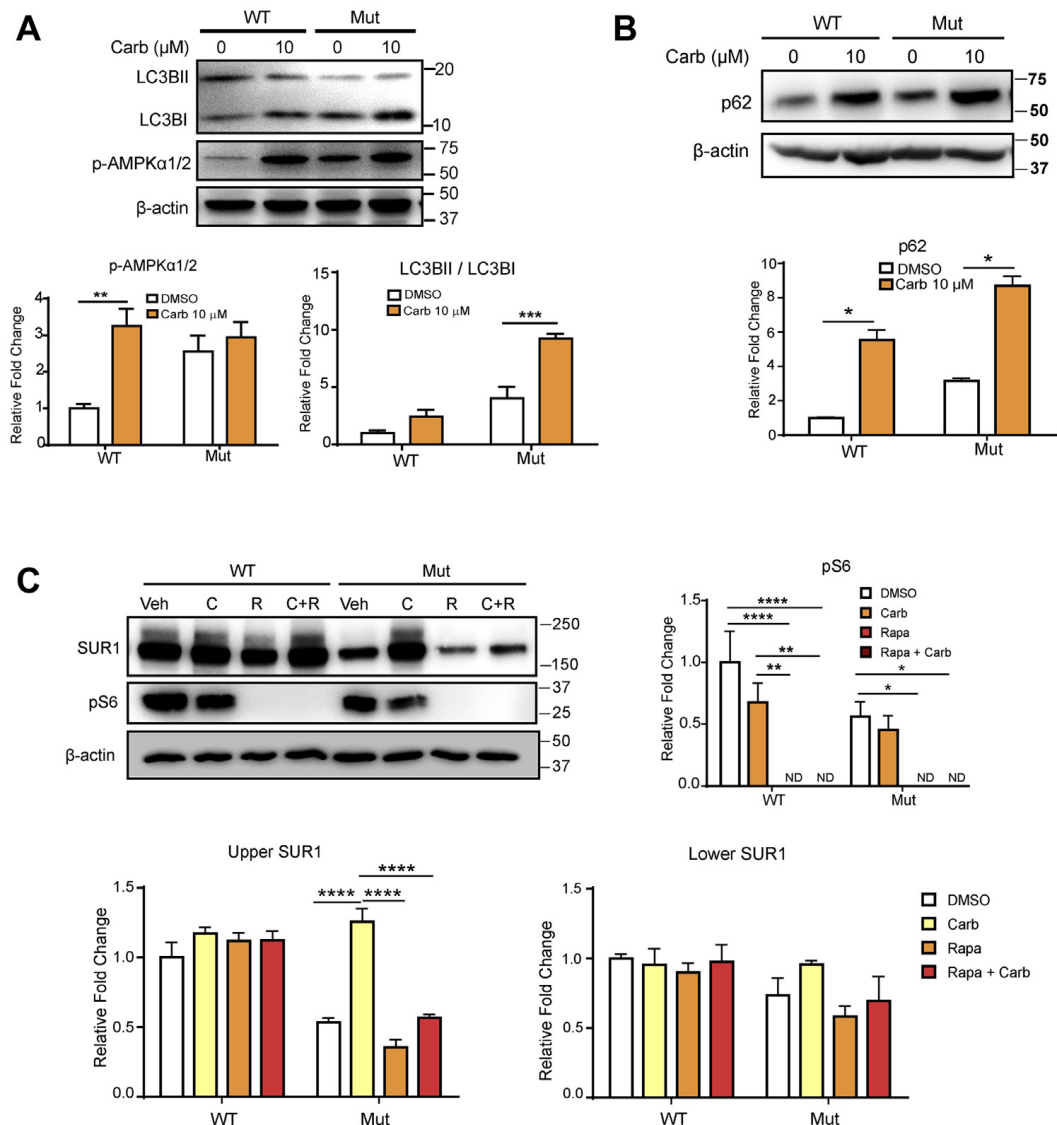
**Carbamazepine (Carb) prevents mutant K**_**ATP**_**channels from autophagy-dependent protein degradation.***A*, *top*, representative immunoblots for LC3BI, LC3BII, and p-AMPKα1/2 in INS-1 cells expressing WT (HA-SUR1/Kir6.2) or mutant (HA-SUR1/Kir6.2-A28V) K_ATP_ channels, following 10 μM Carb treatment for 16 h. *Bottom*, the bar graphs show quantification of the LC3BII/LC3BI ratio and p-AMPKα1/2. *B*, *top*, representative immunoblots for p62 in INS-1 cells expressing WT (HA-SUR1/Kir6.2) or mutant (HA-SUR1/Kir6.2-A28V) K_ATP_ channels, following 10 μM Carb treatment for 16 h. *Bottom*, the bar graphs show quantification of p62. *F*, *top*-*left*, representative immunoblots show the levels of pS6 and SUR1 in INS-1 cells expressing WT or mutant K_ATP_ channels, after treatment with vehicle, 10 μM Carb, 25 μM rapamycin, or 25 μM rapamycin + 10 μM Carb for 16 h. *Top*-*right*, the bar graph shows the levels of pS6. *Bottom*, quantification of the upper (mature) and lower bands of SUR1 is shown in the *left* and *right* plots, respectively. Data are presented as the mean ± SEM; n = 3 per group; ∗*p* < 0.05, ∗∗*p* < 0.01, ∗∗∗∗*p* < 0.001, compared with the WT-vehicle group; differences were evaluated using two-way ANOVA and Dunnett's post hoc test. AMPK, AMP-activated protein kinase; HA, hemagglutinin; K_ATP,_ ATP-sensitive potassium channel; SUR1, sulfonylurea receptor 1.

We then wanted to test whether inhibition of lysosomal or proteasomal degradation could increase the surface expression of mutant K_ATP_ channels. Thus, we treated the channel-expressing INS-1 cells either with 10 μM of autophagy inhibitor (3MA) and chloroquine (CQ) or proteasome blocker (MG132). We found that similar to Carb, 3MA, CQ, and MG132 could increase the amount of mature K_ATP_ channel (upper band of SUR1), suggesting that the autophagy or proteasome pathway is involved in degrading the mutant K_ATP_ channels (Fig. 7). Carb has been reported as an autophagy-enhancing drug in the liver disease (17); hence, we continued examining the role of autophagy in regulating mutant K_ATP_ channel trafficking; we tested whether rapamycin, an autophagy activator, could prevent the Carb rescue effect. Moreover, it has been shown earlier that in the hypothalamus, the K_ATP_ channel is under the regulation of the mammalian target of rapamycin (mTOR) signaling pathway (18). We found that although Carb could increase the upper SUR1 band, this effect was abolished by rapamycin without affecting overall SUR1 expression (lower SUR1 band) (Fig. 8). These results further strengthen the idea that Carb rescues mutant K_ATP_ channel trafficking by inhibiting the autophagy pathway. During autophagy, the cytosolic form of LC3B (LC3BI) binds phosphatidylethanolamine to form the LC3B–phosphatidylethanolamine-conjugated complex (LC3BII), which will bind with p62 substrate and promote the formation of autophagosome membranes. Thus, AMP-activated protein kinase (AMPK) phosphorylation triggers autophagy, and the ratio of LC3BII to LC3BI represents the level of autophagic activity. In addition, the level of p62 degradation indicates the completion of autophagic flux (19). We, therefore, probed this process using LC3B, p-AMPKα1/2, and SQSTM1/p62 antibodies. Carb treatment increased the ratio of LC3BII/LC3BI and p-AMPKα1/2 in WT or Kir6.2-A28V-expressing cells (Fig. 8*A*). However, Carb treatment did not reduce p62 expression, suggesting that the autophagy process was not completed (Fig. 8*B*). Next, we examined whether Carb treatment could affect mTOR-dependent autophagy using rapamycin as an mTOR inhibitor and probing mTOR-downstream S6 phosphorylation. Carb treatment significantly decreased mTOR-downstream S6 phosphorylation in both WT and mutant K_ATP_ channel–expressing cells, which coincided with the increased surface expression of SUR1 (upper band). Our data further showed that cotreatment of Carb and rapamycin did not restore S6 phosphorylation. We also quantified the upper and lower SUR1 bands and found that Carb can rescue the upper SUR1 band level, but rapamycin treatment had no effect on SUR1 expression levels in mutant K_ATP_ channel–expressing cells. However, Carb could not attenuate the induction of autophagy by rapamycin, which would account for the undetectable upper SUR1 band in cotreated cells (Fig. 8*C*). Collectively, these data suggest that Carb initiates autophagic flux, but the fusion of the autophagosome with the lysosomes is not completed, potentially explaining how Carb treatment could cause decreased localization of mutant K_ATP_ channels in lysosomes.

### Carb and sulphonylureas have the same impact on Kir6.2-A28V K_ATP_ channel trafficking

**Figure 9:**
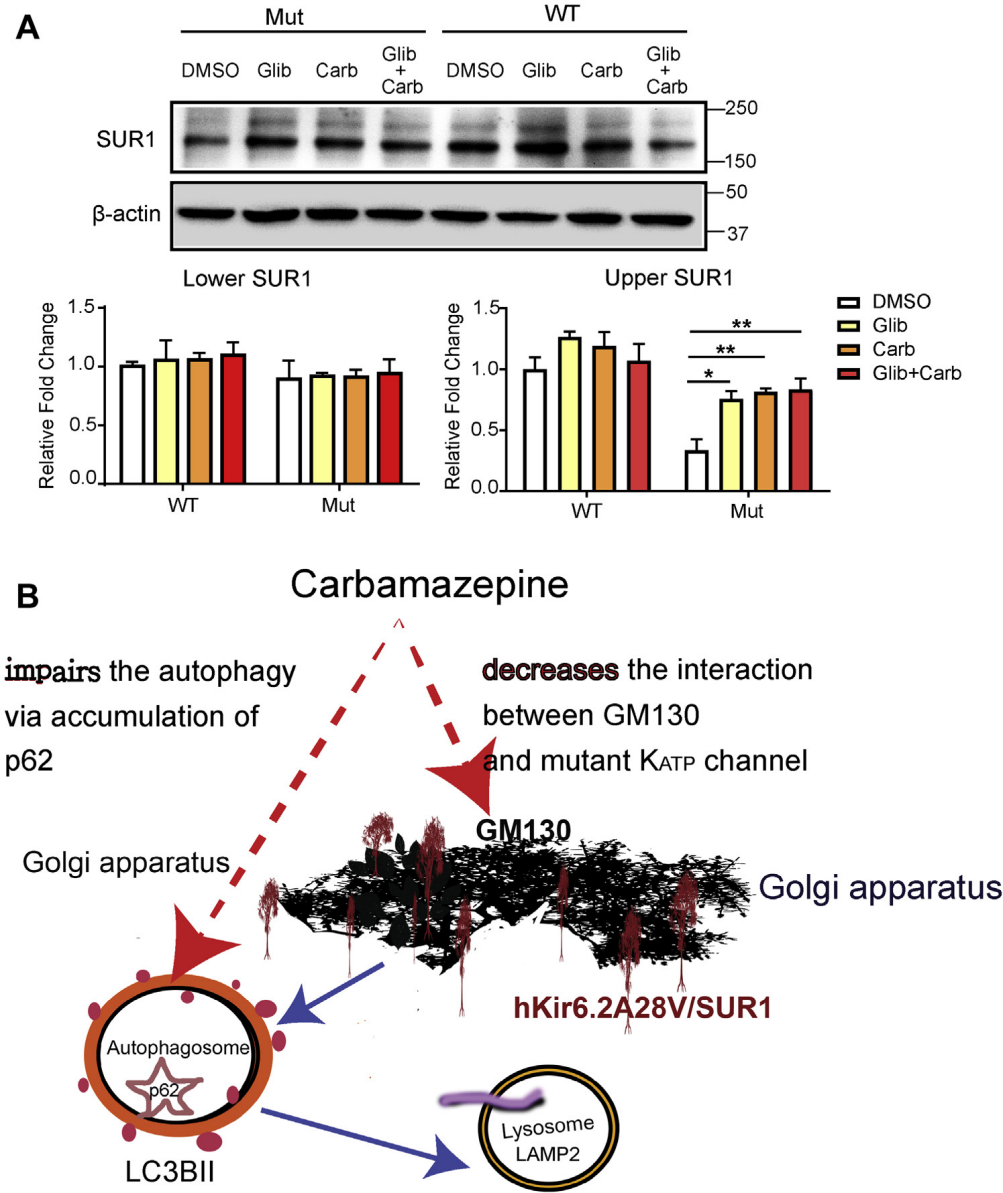
**Carbamazepine (Carb) and sulphonylureas share the same binding site on SUR1.***A*, *top*, representative immunoblots for SUR1 in INS-1 cells expressing WT (HA-SUR1/Kir6.2) or mutant (HA-SUR1/Kir6.2-A28V) K_ATP_ channels, upon treatment with DMSO, 10 μM Glib, or 10 μM Carb +10 μM Glib for 16 h. *Bottom*, quantification of the upper (mature) and lower bands of SUR1 is shown in the *left* and *right* plots, respectively. Data are presented as the mean ± SEM; n = 5 per group; ∗*p* < 0.05, ∗∗*p* < 0.01, ∗∗∗∗*p* < 0.001, compared with DMSO-treated cells in each corresponding group; differences were evaluated using two-way ANOVA and Dunnett's post hoc test. *B*, summary of Carb rescue of mutant K_ATP_ channel trafficking *via* two pathways. First, Carb decreases the interaction of mutant K_ATP_ channels with the Golgi matrix protein, GM130, allowing the mutant channels to reach the cell surface. Second, Carb attenuates autophagy-dependent protein degradation (indicated by accumulation of p62), which allows mutant K_ATP_ channels to pass through the ER. DMSO, dimethyl sulfoxide; ER, endoplasmic reticulum; HA, hemagglutinin; K_ATP_, ATP-sensitive potassium channel; SUR1, sulfonylurea receptor 1.

A recent cryo-EM study demonstrated that Carb and sulphonylureas bind to a common pocket located just above the first nucleotide-binding domain on SUR1 (11). We hypothesized that if these two pharmacochaperones share the same binding site on K_ATP_ channels, cotreatment of Carb and Glib will not produce an additive effect. Indeed, the cotreatment of Carb and Glib increased the upper SUR1 band to a level comparable to single compound treatment alone (Fig. 9*A*). This observation is consistent with the idea that these two pharmacochaperones share the same binding site on SUR1 subunits.

## Discussion

Carb is an antiepilepsy drug used to treat focal and generalized convulsive seizures, especially complex types. The therapeutic mechanism involves drug binding to the inactive form of sodium channels, which reduces the propagation of abnormal impulses while potentiating the action of gamma-aminobutyric acid (20). In addition, Carb may also be used for its antidepressant and analgesic effects (20, 21). The therapeutic range for clinical use is 4 to 12 mg/l (16.9–50.8 μM); so the 10 μM Carb treatment used in our experiments falls within a physiologically relevant range.

Mutations that cause loss of K_ATP_ channel function mostly result in severe types of PHHI that do not respond to the first-line drug, diazoxide, and involve the uncontrolled release of insulin (22). These mutations also often cause defects in channel biogenesis and trafficking, meaning low amounts of K_ATP_ channels are expressed on the plasma membrane. Sulfonylureas, such as Glib or tolbutamide, are used in the management of type 2 diabetes or neonatal diabetes and can affect the K_ATP_ channel response to MgADP (7). In fact, Glib is considered to be a chemical chaperone since it corrects K_ATP_ channel trafficking deficits (8). However, because of the high affinity of Glib binding to K_ATP_ channels, the mutant K_ATP_ channels restored to the cell surface are unresponsive to MgADP stimulation (23, 24). On the other hand, tolbutamide is a low-affinity and reversible sulfonylurea that has been shown to rescue some trafficking mutations and does not affect the channel response to MgADP upon drug washout (9, 25, 26).

Carb may serve as a better chemical chaperone to rescue trafficking mutant K_ATP_ channels. Once the rescued mutant K_ATP_ channels travel to the plasma membrane, they can exert normal functions after Carb washout (Fig. 2) (8, 9, 10, 27). Cryo-EM studies on K_ATP_ channels revealed that Glib and Carb bind within the same pocket of SUR1. This observation suggests a common mechanism by which diverse compounds could stabilize the Kir6.2 N terminus within the SUR1 core, allowing it to act as a firm “handle” for the assembly of metastable mutant SUR1–Kir6.2 complexes (11). We found that Carb corrects the trafficking deficits and increases the surface expression of Kir6.2-A28V-containing K_ATP_ channels. Interestingly, a previous biochemical study also showed that Carb shares the same binding site with Glib, in agreement with the cryo-EM findings (11).

It has been reported that Carb acts as an autophagy enhancer *via* an mTOR-independent pathway and decreases inositol and inositol trisphosphate receptor signaling (28). The decreased inositol trisphosphate suppresses the Ca^2+^ release from the ER and ATP production, which activates AMPK and induces autophagy (29). In this study, we showed that Carb initiates autophagy, according to the increased recruitment of LC3B and AMPK phosphorylation as well as the lack of reduced levels of the mTOR-downstream substrate, pS6. Carb treatment also led to an accumulation of p62. This result was surprising since the p62 protein recognizes cellular waste designated for degradation during the process of autophagosome fusion with lysosomes (30). Hence, Carb did not appear to stimulate the last step of autophagy, explaining the rescue effect on Kir6.2-A28V mutant K_ATP_ channels. We, therefore, speculate that Carb might rescue the surface expression of mutant K_ATP_ channels at least in part by blocking or reducing the degradation of mutant K_ATP_ channels.

Both SUR1 and Kir6.2 contain tripeptide (RKR) ER-retention sequences, which become masked only after proper assembly of all eight subunits. Thus, proper intersubunit interactions are required for efficient channel biogenesis (4). Elimination of the RKR ER-retention signal by mutation to AAA improves the expression of some mutants (31, 32, 33); however, this approach is not feasible as a clinical therapy. On the other hand, chemical chaperones are promising clinical tools. Several mutations that reduce the expression of Kir6.2 and SUR1 can be rescued by treatment with sulfonylureas (25, 34). With regard to Kir6.2-A28V mutant K_ATP_ channels, *de novo* protein structure prediction showed that the mutation reorients the RKR motif, potentially causing trafficking deficits (3). However, in our experiments, we found that Kir6.2-A28V mutant K_ATP_ channels seem to escape from the ER quality control and can still be transported to the Golgi apparatus.

Emerging evidence suggests that GM130 is centrally involved in the control of glycosylation and transport of proteins in the secretory pathway (35). Interestingly, a previous study has identified a novel role of N-linked glycosylation in forward trafficking, internalization, and degradation of the Kv1.2 channel (36). We used co-IP to identify a physiologically relevant interaction between Kir6.2-A28V mutant K_ATP_ channels and GM130. This observation leads us to speculate that elongation of N-glycans in the Golgi apparatus may fail on the mutant channels. Carb treatment decreased the interaction between GM130 and Kir6.2-A28V mutant K_ATP_ channels, and this reduced interaction corresponded to improvements in Kir6.2-A28V mutant K_ATP_ channel trafficking to the cell surface. The importance of GM130 in this process is reminiscent of a previous report showing that the cytoplasmic C terminus of HERG (human Ether-à-go-go-Related Gene)-encoded potassium channel participates in the tethering or possibly targeting of HERG-containing vesicles within the Golgi *via* its interaction with GM130 (37).

Previous publications have shown that Carb can rescue the surface expression of several SUR1 mutants (9, 25, 26, 38). Our study extends these findings to show that Kir6.2-A28V mutant K_ATP_ channels can also respond well to Carb treatment. As such, many other Kir6.2 mutants await testing (39, 40, 41). In terms of mechanism, we showed that in the absence of Carb treatment, expression of the Kir6.2-A28V mutant K_ATP_ channel triggers autophagy, whereas expression of WT K_ATP_ channels does not. Carb treatment blocks the fusion of lysosomes and autophagosomes, according to the observed accumulation of p62. Thus, autophagy appears to be impaired in mutant K_ATP_ channel–expressing cells. Simultaneously, Carb treatment reduces the interaction of Kir6.2-A28V mutant K_ATP_ channel with GM130, allowing the channel to exit from the Golgi apparatus (Fig. 9*B*). Together, these findings reveal a novel mechanism by which Carb, a pharmacochaperone, promotes the surface expression of Kir6.2-A28V mutant K_ATP_ channel.

## Experimental procedures

### Chemicals and antibodies

Glibenclamide (catalog no.: G0639) was purchased from Research Biochemicals International; N1-(β-d-ribofuranosyl)-5-aminoimidazole-4-carboxamide (catalog no.: A9978), 5H-dibenz [b,f] azepine-5-carboxamide (Carb) (catalog no.: C8981), 3-methyladenine (catalog no.: M9281), CQ phosphate (catalog no.: PHR1258), MG132 (catalog no.: M8699), and rapamycin (catalog no.: R8781) were purchased from Sigma–Aldrich. p-AMPKα1/2 (Thr 172) (catalog no.: SC-33524; Research Resource Identifier [RRID]: AB_2169714) antibody was purchased from Santa Cruz Biotechnology; GM130 (catalog no.: ab52649; RRID: AB_880266) and KDEL (catalog no.: ab2898; RRID: AB_303392) antibodies were purchased from Abcam; actin (catalog no.: A5441; RRID: AB_476744) antibodies were purchased from Sigma–Aldrich. HA tag (catalog no.: 26183; RRID: AB_10978021) antibody was purchased from Invitrogen. SUR1 (catalog no.: MABN501; RRID: AB_11001671) was purchased from Merck. Phospho-S6 ribosomal protein (Ser235/236) (catalog no.: 4858; RRID: AB_916156) and LC3B (catalog no.: PA5-32254; RRID: AB_2549727) antibodies were purchased from Cell Signaling. Secondary antibodies: Alexa 488 goat anti-rabbit immunoglobulin G (IgG) (H + L) (catalog no.: A-11008; RRID: AB_143165), Alexa 555 goat anti-rabbit IgG (H + L) (catalog no.: A-21428; RRID: AB_2535849), and Alexa 555 goat antimouse IgG (H + L) (catalog no.: A-21422; RRID: AB_2535844) were purchased from Invitrogen. Sheep antimouse IgG (catalog no.: NA931; RRID: AB_772210) and goat anti-rabbit IgG (catalog no.: NA934; RRID: AB_772206) were purchased from GE Healthcare.

### Cell culture and transfection

The complementary DNAs (cDNAs) for human Kir6.2 (hKir6.2), human SUR1 (hSUR1), HA-tagged rodent SUR1 (HA-SUR1), and Kir6.2-A28V were subcloned into pcDNA3 plasmids (3). Rat INS-1 cells were cultured in RPMI1640 medium (catalog no.: 31800089; Gibco) containing 11 mM glucose and supplemented with 10% fetal bovine serum (catalog no.: 10437-028; Gibco), 1% penicillin–streptomycin (catalog no.: 01161018; Caisson Labs), 2 mM glutamine, 1 mM sodium pyruvate (TCL015; HiMedia), 10 mM Hepes (catalog no.: CC519-0100; GeneDireX), and 50 μM β-mercaptoethanol (catalog no.: 21985-023; Gibco) at 37 °C with 5% CO_2_. HEK293 cells were cultured in Dulbecco's modified Eagle's medium with 10% fetal bovine serum, 1% penicillin–streptomycin, and 0.01% mycoplasma inhibitor. For a 6-well plate, each well (6 × 10^5^ cells) was transfected with 0.3 μg hSUR1 (HA-SUR1) and 0.2 μg Kir6.2 (either WT or A28V) using FuGENE HD (catalog no.: E2311; Promega). Cells were cultured for 40 h at 37 °C before treatment with different compounds for 16 h.

### Whole-cell patch-clamp recording in HEK cells

hKir6.2 and hSUR1 (Origene) cDNAs were cloned into the pcDNA3 plasmid as described previously (3). HEK293 cells were cultured in Dulbecco's modified Eagle's medium (Thermo Fisher Scientific) containing 10% fetal bovine serum (Hyclone), 2 mM glutamine, 100 units penicillin, and 100 mg/ml streptomycin in a humidified atmosphere of 5% CO_2_ at 37 °C. Cells were plated on poly l-ornithine-coated glass coverslips and transiently transfected with 0.2 μg of the pcDNA3 containing hKir6.2 construct and 0.8 μg of pcDNA3 containing SUR1 construct by using PolyJet (SignaGen). Cells were used 2 to 4 days after transfection. K_ATP_ currents were recorded using the whole-cell patch-clamp configuration by an Axopatch 700B amplifier (Molecular Device) in a standard extracellular solution containing 150 mM NaCl, 10 mM Hepes, 5 mM KCl, 2 mM CaCl_2_, 1 mM MgCl_2_, and pH 7.2, adjusted with NaOH. Data were acquired at 10 kHz with pCLAMP software (Molecular Devices). Pipettes were pulled from 1.5 mm borosilicate glass capillaries (Sutter, Inc). Pipette resistances were 2 to 4 MΩ when filled with the intracellular solution containing 135 mM potassium gluconate, 15 mM KCl, 10 mM Hepes, 0.5 mM Mg_2_ATP, 1 mM Na_3_GTP, 10 mM sodium phosphocreatine, 0.05 mM EGTA, and pH 7.2, adjusted with KOH. The access resistances of whole-cell recording ranged between 5 and 20 MΩ and were compensated by ∼80%. All experiments were performed at room temperature (∼25 °C).

### Immunofluorescence staining

HEK293 cells transfected with HA-SUR1/Kir6.2 were fixed with 4% paraformaldehyde for 15 min and then stored in PBS. Cells were first permeabilized by incubating in PBS with 0.1% Triton X-100 at room temperature for 15 min, followed by washing three times with PBS. Cells were incubated in blocking solution (3% bovine serum albumin [BSA] in PBS) with primary antibodies at 4 °C overnight and then with secondary antibodies in PBS for 1 h at room temperature. After washing three times with PBS, the cells were incubated in Hoechst dye (1:10,000 in PBS) for 10 min. A mounting medium for fluorescence was used to mount the coverslips on the slides. Images were visualized by epifluorescence or confocal microscopy. For surface staining, cells were washed three times in cold PBS and then incubated in anti-HA antibody (1:200 in Opti-MEM with 0.1% BSA) for 1 h at 4 °C. After washing out the primary antibody, cells were fixed with ice-cold methanol for 10 min on ice and then washed three times in cold PBS, followed by incubation in blocking buffer (PBS with 3% BSA) for 1 h at room temperature. Cells were then incubated with the secondary antibodies for 1 h. Hoechst staining and coverslip mounting were performed as described previously.

### PLA

The samples for immunofluorescence staining were prepared according to previously described immunohistochemistry staining (42). We used Duolink Blocking Solution to cover the entire glass slides and then incubate the glass slides in a heated humidity chamber for 1 h at 37 °C. Following, anti-Kir6.2 and anti-GM130 antibody dilute in Duolink Antibody Diluent and then incubate overnight at 37 °C. After removing the primary antibody and washing twice, the glass slides were incubated with the minus and the plus probe (1:5 in the Duolink Antibody Diluent) for 1 h at 37 °C. After washing twice with wash buffer A, we added ligase to the 1× ligation buffer (1:40 in the Duolink Antibody Diluent) and incubated 30 min at 37 °C. Following washing two times, we added polymerase to the 1× amplification buffer (1:80) and then incubated for 100 min at 37 °C (also all solutions containing buffer were protected from light). Wash slides 2× 10 min in 1× wash buffer B and then 0.01× wash buffer B for 1 min. Finally, we mounted the slides with a coverslip using a minimal volume of Duolink *In Situ* Mounting Medium with 4′,6-diamidino-2-phenylindole for 15 min.

### Immunoprecipitation

NeutrAvidin-agarose beads (catalog no.: 29200; Thermo Fisher Scientific) were incubated with 300 μg protein samples on a rotator overnight at 4 °C. Unbound proteins were washed away with lysis buffer (20 mM Hepes, 150 mM NaCl, 4 mM EDTA, 1 mM EGTA, 1% Igepal, 0.1% SDS, and 0.04% deoxycholic acid with cOmplete protease inhibitors, pH adjusted to 7.2). To elute the bound proteins, 2× sample buffer was mixed with the beads for 30 min at room temperature. Immunoblotting was performed to detect protein expression. For co-IP, cell lysates were incubated with 2 μg of antibody and protein A-Sepharose beads (catalog no.: ab193256; Abcam) for 4 h at 4 °C. The immunocomplexes were then washed with triple lysis buffer three times and separated by SDS-PAGE. Immunoblotting was performed according to standard procedures.

### mRNA expression analysis

TRIzol (catalog no.: AM9738; Ambion) was used to isolate RNA. The RNA was purified using chloroform, then precipitated with isopropanol, and washed with 70% EtOH. The abundance and quality of isolated RNA was determined spectroscopically (NanoDrop ND-1000; Thermo Fisher Scientific). One microgram purified RNA was reverse transcribed using the MMLV Reverse Transcription Kit (catalog no.: PT-RT-KIT; Protech Technology Enterprise). The mRNA expression levels were determined using quantitative real-time PCR with Smart Quant SYBR Green Master Mix with ROX (catalog no.: PT-GL-SQGR-V3-1ML; Protech Technology Enterprise). The primers used in this study are listed in Table1. Ribosomal 18s RNA was used as an internal control. The 2^−ΔΔCt^ method was used for mRNA quantification.

**Table 1:** Primers used for q-PCR

Target gene	Forward primer	Reverse primer
IRE1α	5′-ACACTGCCTGAGACCTTGTTG-3′	5′-GGAGCCCGTCCTCTTGCTA-3′
PERK	5′-CGCGTCGGAGACAGTGTTT-3′	5′-GTCCTCCACGGTCACTTCG-3′
ATF6α	5′-TCGCCTTTTAGTCCGGTTCTT-3′	5′-GGCTCCATAGGTCTGACTCC-3′
HRD1	5′-CTAGGACCTTGTCCTTTGGGG-3′	5′-CAGAGACCTGTGAACGCTAGG-3′
18s RNA	5′-AGACGGACCAGAGCGAAAG-3′	5′-CCAGTCGGCATCGTTTATG-3′

### Western blot

HEK293 cells were lysed by incubating in triple lysis buffer for 15 min, followed by sonication and centrifugation at 16,000*g* for 20 min at 4 °C. The supernatants were collected, separated by 7% or 12% SDS-PAGE, and then transferred onto polyvinylidene difluoride membranes. The membranes were incubated with primary antibodies overnight at 4 °C. Horseradish peroxidase–conjugated secondary antibodies were applied for 1 h at room temperature. Signals were detected using an enhanced chemiluminescence detection kit (catalog no.: 32106; Thermo Fisher Scientific).

### Surface biotinylation

HEK293 cells were washed with cold Dulbecco's PBS (dPBS) three times, and the surface proteins were then biotinylated by incubating with 1 mg/ml EZ-Link Sulfo-NHS-SS-Biotin (catalog no.: 21331; Thermo Fisher Scientific) dissolved in dPBS for 30 min. Cells were incubated in 20 mM glycine for 5 min twice, then washed with dPBS three times to terminate the reaction, and cells were lysed with triple lysis buffer for 30 min. The procedure was performed on ice to prevent protein denaturation. Samples were collected using the same protocol as used for immunoblotting.

### Microscope examination

All images were visualized by confocal laser scanning microscopy Olympus FV300.

### Statistical analysis

All data were analyzed using GraphPad Prism 6 (GraphPad Software, Inc). Results are expressed as mean ± SEM. Differences were tested using either one-way or two-way ANOVA followed by the post hoc Dunnett's test for multiple comparisons. The level of statistical significance was set at *p* < 0.05.

## Data availability

All data presented in this article are available upon request.

## Supporting information

This article contains supporting information.

## Conflict of interest

The authors declare that they have no conflicts of interest with the contents of this article.
