# Supplementary material for: Carbamazepine promotes surface expression of mutant Kir6.2-A28V ATP-sensitive potassium channels by modulating Golgi retention and autophagy
Source: J Biol Chem. 2022 Apr 6;298(5):101904. doi: 10.1016/j.jbc.2022.101904 (PMC9065613; doi:10.1016/j.jbc.2022.101904)
Supplement: Supplemental Figure S1 [file mmc1.pdf]

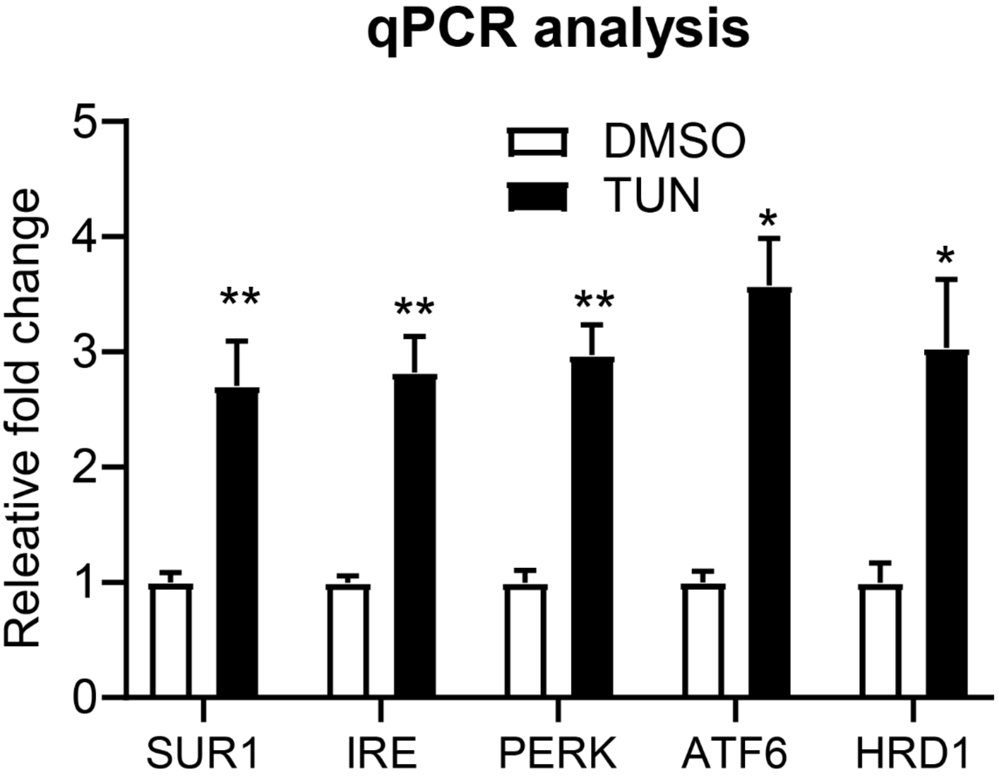

Suppl Fig. 1

Suppl Fig. 1 Tunicamycin activates UPR or ERAD. Relative expression levels of SUR1 mRNA in INS-1 cells treated with tunicamycin (1.25 μg/mL) for 12h. The relative expression of SUR1, key UPR sensors (i.e., IRE1, PERK, ATF6) and ERAD sensor, Hrd1, were increased as compared to DMSO treated INS-1 cells. Data are presented as the mean ± SEM; n = 6 per group; no results achieved significance when compared with DMSO-treated cells in each corresponding group; differences were evaluated using two-way ANOVA and Dunnett's post hoc test.
